# CRISPR/Cas9 mediated mutation of mouse IL-1α nuclear localisation sequence abolishes expression

**DOI:** 10.1038/s41598-017-17387-x

**Published:** 2017-12-06

**Authors:** Michael J. D. Daniels, Antony D. Adamson, Neil Humphreys, David Brough

**Affiliations:** 1 Division of Neuroscience and Experimental Psychology, School of Biological Sciences, Faculty of Biology, Medicine and Health, Manchester Academic Health Science Centre, University of Manchester, AV Hill Building, Oxford Road, Manchester, M13 9PT UK; 2 Transgenic unit, Faculty of Biology, Medicine and Health, University of Manchester, AV Hill Building, Oxford Road, Manchester, M13 9PT UK

## Abstract

Inflammation is a host defense process against infection. Inflammatory mediators include cytokines of the interleukin-1 family, such as IL-1α and IL-1β. Unlike IL-1β, IL-1α carries an N-terminal nuclear localisation sequence (NLS) and is trafficked to the nucleus. The importance of IL-1α nuclear localisation is poorly understood. Here, we used CRISPR/Cas9 to make inactivating mutations to the NLS on the *Il1a* gene. A colony of NLS mutant mice was successfully generated with precise knock-in mutations to incapacitate NLS function. NLS mutant mice had no gross changes in immunophenotype or inflammatory response but, surprisingly, failed to express IL-1α. We deduced that, in making specific mutations in the *Il1a* gene, we also mutated a long-noncoding (lnc)RNA in the complementary strand which has cis-regulatory transcriptional control of the *Il1a* gene itself. The mutations generated in the *Il1a* gene also result in mutation of the lncRNA sequence and a predicted alteration of its secondary structure, potentially explaining a subsequent failure to function as a transcriptional activator of *Il1a* expression. Thus, lncRNA secondary structure may regulate IL-1α expression. Our results serve as a cautionary note that CRISPR –mediated genome editing without full knowledge of genomic context can result in unexpected, yet potentially informative observations.

## Introduction

Inflammation is generally a protective host response to injury and infection. Inflammation is initiated by detection of either ‘pathogen’ or ‘damage’-associated molecular patterns by pattern recognition receptors and subsequent secretion of pro-inflammatory cytokines which amplify the inflammatory response by recruiting immune cells to the site of injury/infection^1^.

The interleukin (IL)-1 family of proteins, the major members of which are IL-1α and IL-β, are amongst the best-studied pro-inflammatory cytokines. IL-1α and β initiate an inflammatory response by binding IL-1 receptor 1 (IL-1R1) which, following recruitment of IL-1R accessory protein, triggers signalling pathways leading to further pro-inflammatory gene transcription^2^.

Inflammation is not always protective, and both IL-1α and IL-1β are involved in the pathogenesis and progression of numerous non-communicable diseases including stroke^3^, diabetes^4^, atherosclerosis^5,6^, Alzheimer’s disease^7,8^, and others^9^. IL-1α and IL-1β are highly regulated. The regulation and secretion of IL-1β, governed by the formation of a multi-molecular complex called an inflammasome, is relatively well researched^10^. However, the mechanisms of IL-1α regulation are poorly understood.

Both IL-1α and IL-1β are synthesised as 31 kDa precursor proteins (pro-IL-1β and pro-IL-1α) in response to a pathogen or damage associated signals, and are secreted from cells via unconventional secretory mechanisms after processing to active 17 kDa forms^11^. IL-1α is also transcriptionally regulated by a long, noncoding RNA (lncRNA) located on the antisense strand of the *Il1a* gene^12^. lncRNAs are noncoding RNA molecules over 200 nucleotides and have a well-established role in regulation of innate immune mechanisms^13^. lncRNAs therefore provide an additional level of regulation for inflammation upstream of protein translation. AS-IL1α, a lncRNA located on the complementary strand of *Il1a* itself, is essential for LPS-induced expression of IL-1α^12^.

IL-1 family cytokines are also regulated by subcellular localisation. Once expressed, pro-IL-1β is evenly distributed across the cytosol, whilst pro-IL-1α is both cytosolic and nuclear^14^. Nuclear enrichment of IL-1α is due to the presence of a nuclear localisation sequence (NLS) within the N-terminus pro-piece^15^. NLSs are short motifs of amino acids that target proteins for active transport through the nuclear envelope by the karyopherin-β (kapβ) family of transport receptors^16^. Classical NLSs comprise short sequences of amino acids characterised by lysine residues and are either monopartite^17^ (a single stretch of amino acids) or bipartite^18^ (two stretches separated by a linker region). The NLS of IL-1α is a highly conserved classical monopartite sequence defined as KVLKKRRL in human and KILKKRRL in mouse^15^.

The importance of nuclear localisation in the regulation of IL-1α is poorly understood. A related IL-1 family member IL-33 requires nuclear localisation for its own sequestration to prevent aberrant damaging inflammation^19^. We have shown previously that NLS function can be abrogated *in vitro* by mutating a single lysine at position 85 using site-directed mutagenesis and that IL-1α can be retained in the nucleus of necrotic cells suggesting the NLS may serve an anti-inflammatory function by inhibiting release^14,20^. Similar studies have also been carried out to suggest that nuclear localisation of IL-1α may be important in cell growth^21–23^, motility^24,25^, cell death^26^ and cytokine secretion^27–29^. However, *in vitro* approaches used in the past are hampered by limitations such as over expression, the use of short constructs rather than full length genes and background expression of endogenous wild type (WT) genes. More accurate approaches are therefore required to assess the role of nuclear localisation of IL-1α.

The ability to target and modify the endogenous version of a gene of interest through gene editing/genome engineering has greatly expanded the range of experimental possibilities and enhanced the accuracy of representative model systems generated. The latest generation of gene editing technology, clustered, regularly interspaced, short palindromic repeat (CRISPR)/Cas9, a re-purposed bacterial adaptive immune system, has rapidly gained popularity owing to its simplicity of application. Targeting of specific genomic regions is achieved through directing a nuclease, Cas9, *via* a short guide RNA sequence (~110 nt) that is easy to design and generate in the laboratory, the only targeting restriction being the requirement of a protospacer adjacent motif (PAM) site downstream of the guide target site. For the most widely used CRISPR system from *Streptococcus pyrogenes* (SpCas9) this motif is NGG, which is common in most genomes. This restriction is further mitigated by the use of engineered PAM targeting mutants^30^ or through the use of CRISPR systems from different bacterial species^31^. CRISPR thus enables us to develop and build more representative biological model systems for experimentation.

CRISPR has been demonstrated to work in most genomes targeted, from bacteria to mammals, and has been applied extensively to the generation of transgenic mouse models^32^. In mammalian genomes, once Cas9 binds to target DNA two nuclease domains, RuvC and HNH, facilitate DNA double strand break (DSB) and the resulting DNA repair mechanisms can be exploited to generate desired mutations and modifications. Many DNA repair mechanisms exist, but with respect to CRISPR, Non-Homologous End Joining (NHEJ) and Homology Directed Repair (HDR) are at present most commonly used. NHEJ can result in the insertion or deletion of a few bases of DNA at the target site, known as InDels, which can easily lead to a frameshift of the gene reading frame and knockout of the gene, or the disruption of transcription factor (TF) binding sites. HDR requires the supply of a DNA repair template component, which contains a specific mutation to be generated flanked by appropriately sized homology arms. Precise modification by HDR can be used to make specific point mutations in target genes^33^.

In this study we used CRISPR/Cas9 to target the coding sequence of the IL-1α gene to mutate the NLS *in vivo* for the first time. However, in doing so we also mutated a lncRNA in the complementary strand resulting in a predicted modification in its structure. Expression of IL-1α was inhibited in the NLS mutant mouse. We propose that this was most likely caused by the corresponding mutation in the lncRNA. Thus, this study demonstrates the potential caveats of CRISPR/Cas9 technology.

## Results

### The NLS of IL-1α can be successfully mutated *in vivo* by CRISPR gene editing

**Figure 1:**
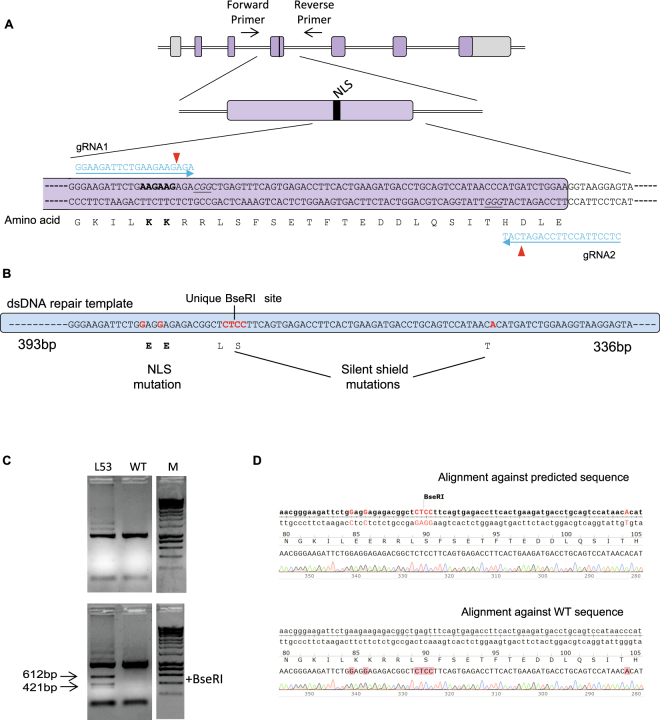
The NLS of IL-1α can be successfully mutated *in vivo* by CRISPR gene editing. Schematic of mouse *Il1a* gene, indicating nuclear localisation sequence (NLS) and CRISPR guide target sites (**A**). Design of the 816 bp dsDNA repair template, note the point mutations to inactivate NLS and shield mutations to both inactivate CRISPR targeting, and create a unique BseRI restriction site for genotyping purposes (**B**). Genotyping of founder 53, the 1033 bp PCR product is digested to 421 bp and 612 bp products. Marker is Hyperladder 1 kb (Bioline) (**C**). Sequence confirmation of mutant allele. Red bases indicate mutations generated by HDR (**D**).

We identified two gRNA target sites in exon 3 of the murine *Il1a* gene close to the residues to be mutated (Fig. 1A). We designed an 816 bp double stranded DNA repair template with 5′ and 3′homology to this region (Fig. 1B), designed to modify the KK NLS motif at position 85/86 to EE. Also included were a further 4 base pair substitutions that function to act as shield mutations (to prevent gRNA/Cas9 re-binding and cutting the repaired region) and generate a unique BseRI restriction site for screening and genotyping purposes. These mutations in the coding region were silent, coding for the same amino acids as in the WT gene (Fig. 1C). Of the 87 pups screened two (animals 53 and 63) were identified with precise knock in of the mutated sequences, and confirmed by sequencing (Fig. 1D). Animal 53 was used to establish a colony of IL1aNLSmut mice after confirmation of germline transmission (animal 63 failed to breed).

### NLS mutant mice have no overt phenotype

**Figure 2:**
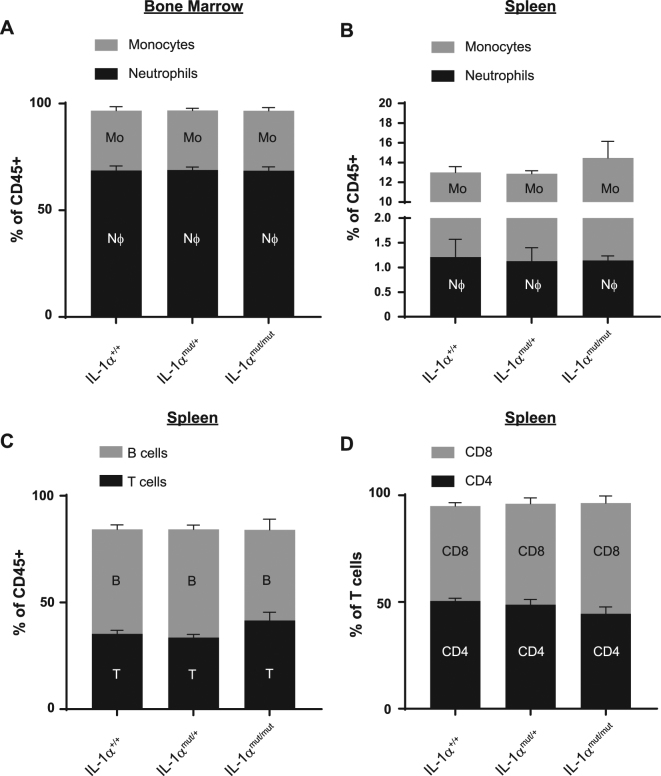
NLS animals have no gross changes in immunophenotype. Single cell suspensions of bone marrow and spleen were isolated from IL-1α^+/+^, IL-1α^mut/+^ or IL-1α^mut/mut^ mice and assessed for major immune cell populations by flow cytometry. Immune populations assessed in bone marrow were Ly6G + neutrophils (Nϕ) and Ly6C + monocytes (Mo) (**A**). Immune populations assessed in spleen were neutrophils and monocytes (**B**), CD19 + B-cells and TCRb + T-cells (**C**), and CD4 + T-cells (CD4) and CD8 + T-cells (CD8) (**D**). Data are presented as mean % cell-type + s.e.m (n = 4), analysed by two-way ANOVA. There were no significant differences between genotype.

We first assessed whether IL-1α NLS mutant mice had any gross immunological differences to their WT counterparts. Age and sex-matched mice either homozygote for the mutant allele (IL-1α^mut/mut^), hetereozygote (IL-1α^mut/+^), or WT (IL-1α^+/+^) were sacrificed and cells taken from the major lymphoid organs, the spleen and the bone marrow. Flow cytometry analysis showed that there was no difference in proportions of neutrophils or monocytes, the major immune cells of the bone marrow, between either IL-1α^mut/mut^ or IL-1α^mut/+^ mice and IL-1α^+/+^ controls (Fig. 2A). Neutrophil and monocyte populations were also evaluated in the spleen and there were no differences observed between genotypes (Fig. 2B). Gross changes in adaptive immunity were assessed by analysis of T and B-cell populations in the spleen and by CD4 and CD8 T-cell subtype (Fig. 2C,D). These data show that there were no changes in basal populations of immune cells in IL-1α^mut/mut^ or IL-1α^mut/+^ mice comparted to IL-1α^+/+^ littermates.

**Figure 3:**
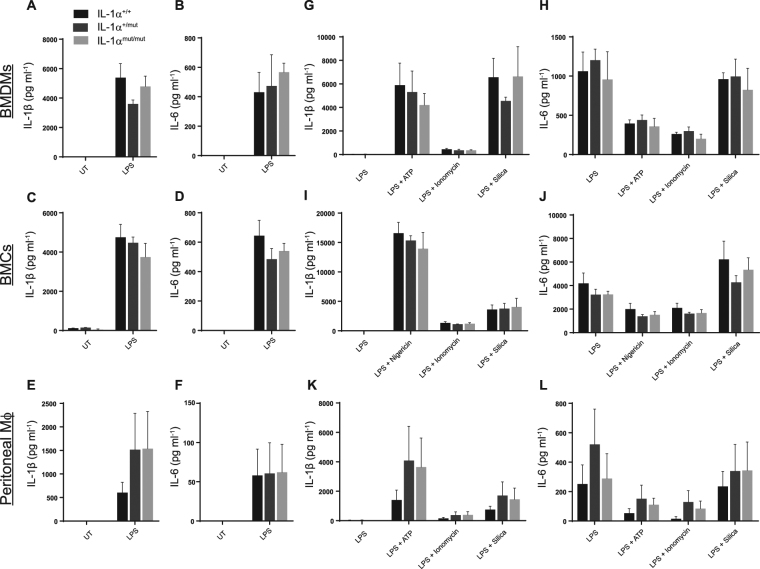
Proinflammatory cytokine production/secretion is not impaired in NLS mutant mice. Immune cells were isolated from IL-1α^+/+^, IL-1α^mut/+^ or IL-1α^mut/mut^ mice and treated with LPS (1 μg ml^−1^, 4 h) before assessing lysates for cytokine production by ELISA. BMDM lysates were assessed for IL-1β (**A**) and IL-6 (**B**) as were lysates taken from BMCs (**C**,**D**) and peritoneal macrophages (**E**,**F**). Immune cells were also stimulated with ATP or nigericin, ionomycin, and silica to promote cytokine secretion. BMDM supernatants were assessed for IL-1β (**G**) and IL-6 (**H**) as were supernatants taken from BMCs (**I**,**J**) and peritoneal macrophages (**K**,**L**). Data are presented as mean IL-1α production + s.e.m (n = 4), analysed by two-way ANOVA. There were no significant differences between genotype.

We next investigated whether cells from IL-1α^mut/mut^ mice responded normally to inflammatory stimuli. We isolated bone marrow-derived macrophages (BMDMs) (Fig. 3A,B), mixed bone marrow cultures (BMCs) (Fig. 3C,D) and peritoneal macrophages (Fig. 3E,F) from IL-1α^+/+^, IL-1α^mut/+^ and IL-1α^mut/mut^ mice and stimulated cells with the bacterial cell wall component lipopolysaccharide (LPS) to induce cytokine production/secretion. Cell lysates were analysed for the expression of pro-inflammatory cytokines IL-1β (Fig. 3A,C,E) and IL-6 (Fig. 3B,D,F) by ELISA. There was no difference in IL-1β or IL-6 expression between IL-1α^mut/mut^ or IL-1α^mut/+^ mice and IL-1α^+/+^ controls. We next tested whether the cellular release of IL-1β and IL-6 was altered in IL-1α^mut/mut^ mice. IL-6 is secreted in response to LPS stimulation alone^34^. However, secretion of IL-1β must be induced by both LPS and a second signal which leads to activation of the NLRP3 inflammasome and caspase-1-dependent proteolytic processing from the 31 kDa pro form into a 17 kDa mature form^35^. BMDMs (Fig. 3G,H), BMCs (Fig. 3I,J) and peritoneal macrophages (Fig. 3K,L) were isolated from IL-1α^+/+^, IL-1α^mut/+^ and IL-1α^mut/mut^ mice and stimulated with LPS alone or primed with LPS and then stimulated with ATP or nigericin, and silica to activate the NLRP3 inflammasome. Cells were also stimulated with the Ca^2+^ ionophore ionomycin, which is known to activate Ca^2+^-dependent processing and release of IL-1α^36^. Supernatants were assessed for IL-6 and IL-1β by ELISA with preferential detection for the mature form of IL-1β. There were no differences between WT and mutants for the release of IL-1β (Fig. 3G,I,K) or IL-6 (Fig. 3H,J,L) across all stimuli. Together, these data show that mutation of the NLS on IL-1α had no effect on either gross immune cell populations or on secretion of classical pro-inflammatory cytokines from multiple immune cell types.

### CRISPR-mediated disruption of the Il1a gene leads to loss of IL-1α expression

**Figure 4:**
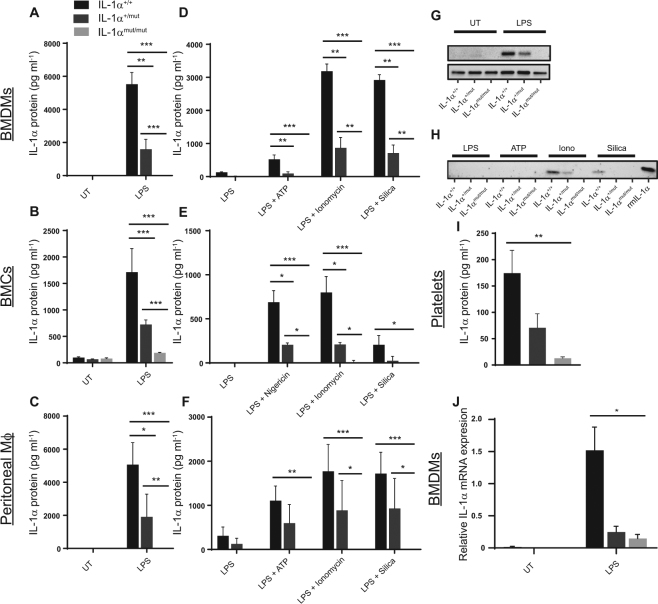
CRISPR-mediated mutation of the *Il1a* gene leads to loss of IL-1α expression. BMDMs (**A**), BMCs (**B**) and peritoneal macrophages (**C**) were isolated from IL-1α^+/+^, IL-1α^mut/+^ or IL-1α^mut/mut^ mice and treated with LPS (1 μg ml^−1^, 4 h) before assessing lysates for IL-1α production by ELISA. BMDMs (**D**), BMCs (**E**) and peritoneal macrophages (**F**) were also stimulated with ATP or nigericin, ionomycin, and silica and supernatants assessed for IL-1α secretion. Data are presented as mean IL-1α production/secretion + s.e.m (n = 3–4) *P < 0.05, **P < 0.01, ***P < 0.01 (two-way ANOVA with repeated measures followed by Tukey’s post-hoc comparison). IL-1α in LPS-stimulated lysates (**G**) and ATP/nigericin/ionomycin/silica-stimulated supernatants (**H**) was measured by western blot. Platelets were isolated from blood of IL-1α^+/+^, IL-1α^mut/+^ or IL-1α^mut/mut^ mice and lysates assessed for IL-1α by ELISA (**I**). Data are presented as mean IL-1α production + s.e.m (n = 6–8) **P < 0.01 (one-way ANOVA with Tukey’s post-hoc comparison). NLS mutation leads to impairments in IL-1α mRNA production. RNA was isolated from BMDMs stimulated with LPS (1 μg ml^−1^, 5 h). IL-1α mRNA was assessed by qPCR (**J**). Data are presented as mean IL-1α expression + s.e.m (n = 4) relative to a reference sample calculated using the standard curve method. *P < 0.05 (two-way ANOVA with repeated measures followed by Tukey’s post-hoc comparison).

We next investigated whether mutation of the NLS of IL-1α had any effect on expression of the IL-1α protein. IL-1α is not normally expressed in monocytes or macrophages and must be induced by a stimulus such as LPS^37–40^. BMDMs, BMCs and peritoneal macrophages were isolated from IL-1α^++^, IL-1α^mut/+^ and IL-1α^mut/mut^ mice and stimulated with LPS as described above. Following treatment, lysates and supernatants were measured for IL-1α protein expression by ELISA and western blot. In BMDMs, LPS stimulation induced production of IL-1α in cells isolated from IL-1α^+/+^ and, to a reduced extent, IL-1α^mut/+^ mice (Fig. 4A). However, LPS treatment was unable to induce IL-1α production in IL-1α^mut/mut^ mice (Fig. 4A). In fact, IL-1α in cells isolated from IL-1α^mut/mut^ mice was completely undetectable. LPS also induced production of IL-1α in IL-1α^+/+^ BMCs, which was again attenuated in IL-1α^mut/+^ BMCs. IL-1α expression in IL-1α^mut/mut^ BMCs was also completely abolished (Fig. 4B). Finally, IL-1α production in peritoneal macrophages was also measured and once again was attenuated in IL-1α^mut/+^ and abolished in IL-1α^mut/mut^ cells (Fig. 4C). We next assessed IL-1α secretion from immune cells stimulated with inducers of the inflammasome, or with Ca^2+^ influx as described above. Secretion of IL-1α was detected in IL-1α^+/+^ and, to a reduced extent, IL-1α^mut/+^ BMDMs (Fig. 4D), BMCs (Fig. 4E) and peritoneal macrophages (Fig. 4F) stimulated with inflammasome activators ATP or nigericin, and silica, and also with the Ca^2+^ ionophore ionomycin. Strikingly, there was no detectable IL-1α released from IL-1α^mut/mut^ cells in response to any of the stimuli tested. Additionally, western blot analysis showed that both LPS-induced expression of 31 kDa pro-IL1α (Fig. 4G) and DAMP-induced secretion of 17 kDa mature IL-1α (Fig. 4H) are attenuated in IL-1α^mut/+^ and abolished in IL-1α^mut/mut^ mice. Full-length blots are presented in Supplementary Figure 1. Cells isolated from IL-1α^mut/+^ mice (and therefore only possessing one WT allele) secreted reduced levels of IL-1α compared to IL-1α^+/+^ cells implying a dose-dependent effect of the allele.

In the above experiments BMDMs, BMCs and peritoneal macrophages were all stimulated with LPS to induce IL-1α production. LPS stimulation leads to IL-1α production via toll-like receptor 4 (TLR4) and NF-κB-mediated transcription^41^. Platelets do not require LPS-stimulation to express IL-1α^42^. We therefore tested whether IL-1α expression in IL-1α^mut/mut^ mice was impaired in platelets. Mouse platelets were isolated from blood and lysed before testing for IL-1α by ELISA. Platelets isolated from IL-1α^+/+^ and IL-1α^mut/+^ mice expressed IL-1α while in IL-1α^mut/mut^ mice IL-1α was barely detectable (Fig. 4I).

We next tested whether abolition of IL-1α expression also occurs at the level of transcription. BMDMs and BMCs were also isolated from IL-1α^+/+^, IL-1α^mut/+^ and IL-1α^mut/mut^ mice and treated with LPS to stimulate transcription of IL-1α mRNA which was measured by quantitative PCR. Production of IL-1α mRNA was upregulated in BMDMs isolated from IL-1α^+/+^ mice following 5 h LPS stimulation (Fig. 4J). However, mRNA production was significantly reduced in BMDMs taken from IL-1α^mut/mut^ mice (Fig. 4J). These data suggest that genetic mutation of the DNA encoding the NLS region of the *Il1a* gene has resulted in loss of both IL-1α mRNA and protein expression.

### Mutation of Il1a does not affect transcription of the crucial regulatory lncRNA AS-IL1α but may affect secondary structure and function

There are multiple feasible explanations for the disruption in IL-1α transcription. One possibility is that the 7 base pair changes made in exon 3 of the *Il1a* gene have prevented binding and recruitment of transcription factors directly to an as yet unidentified autoregulatory exonic enhancer. On rare occasions exons can contain enhancer regions important for expression of nearby genes^43^. However, such eExons typically regulate neighbouring genes - not those in which the eExons sit. We have observed that there is no effect on the expression of the neighbouring gene IL-1β (Fig. 3). It is therefore more likely that this effect is due to other factors controlling IL-1α expression.

**Figure 5:**
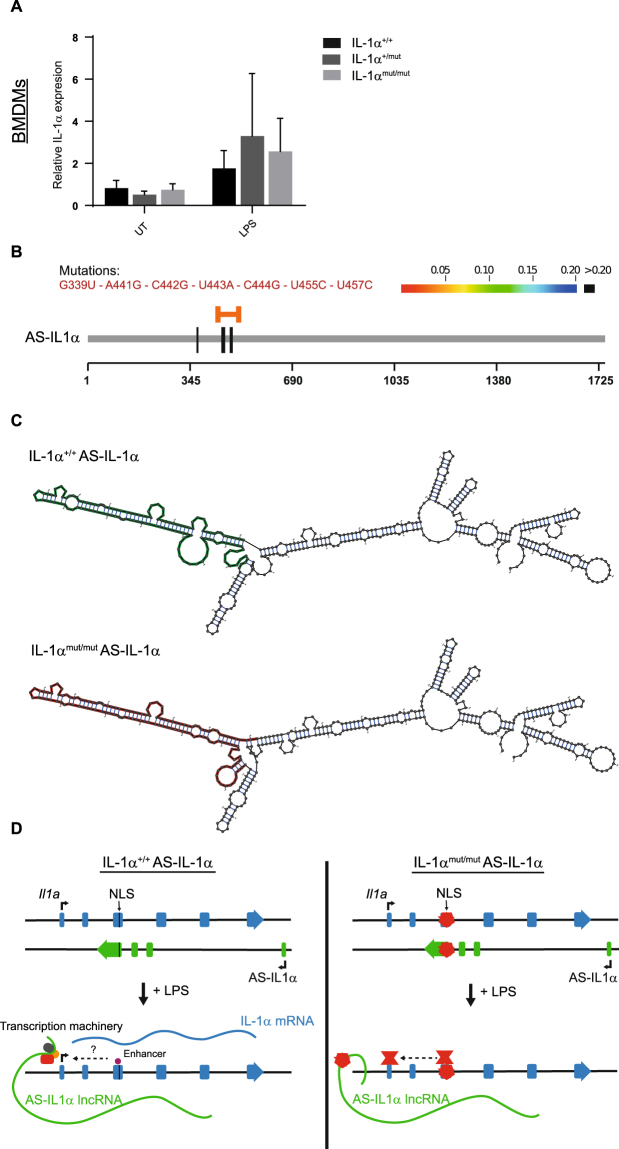
NLS mutation does not affect transcription of AS-IL1α lncRNA but may affect secondary structure and function. AS-IL1α RNA isolated from IL-1α^+/+^, IL-1α^mut/+^ or IL-1α^mut/mut^ BMDMs treated with LPS (1 μg ml^−1^, 5 h) was assessed by qPCR. Data are presented as mean AS-IL-1α expression + s.e.m (n = 4) relative to a reference sample calculated using the standard curve method (**A**). CRISPR-mediated mutations may affect lncRNA structure and function. RNAsnp analysis showed significant structural change in RNA structure located around the mutations made (mutations indicated by vertical black lines, area of significant change indicated by horizontal orange bar) (**B**). Graphical display of AS-IL1α secondary structure shows predicted disruption of RNA loops from WT (green) to mutant (red) sequence, p = 0.0294 calculated by RNAplfold algorithm (**C**). A schematic outlining the proposed mechanism(s) by which the mutation in the *Il1a* gene leads to loss of IL-1α expression. In the wild-type gene (left) LPS induces transcription of the AS-IL1α which, in turn, promotes recruitment of transcriptional machinery to the promoter region of the *Il1a* gene and induces production of IL-1α mRNA. Transcription may also be induced by an enhancer region in exon 3 of the DNA. In the mutant gene LPS induces production of the mutant lncRNA which, due to changes in secondary structure, is unable to recruit transcriptional machinery and thus there is no production of IL-1α mRNA (**D**).

It has been recently shown that transcription of IL-1α in immune cells is controlled by a natural antisense transcript (NAT)^12^. Antisense IL-1α (AS-IL1α) is a long non-coding RNA (lncRNA) encoded on the antisense strand within the *Il1a* locus. Expression of AS-IL1α is induced by LPS and is required in order to promote IL-1α transcription^12^. We hypothesized that the loss of IL-1α expression observed above may be as a result of impaired expression or function of AS-IL1α due to inadvertent genetic perturbation, which in turn prevents induction of IL-1α itself. Thus we measured by qPCR the expression of AS-IL1α in LPS-treated immune cells from IL-1α^mut/+^ or IL-1α^mut/mut^ mice compared to IL-1α^+/+^ littermates and detected no significant difference in expression levels across all three mouse models (Fig. 5A).

Next we investigated potential AS-IL1α lncRNA structural changes generated by the CRISPR induced base mutations. lncRNAs are highly sensitive to structural change and function often relies on secondary structure^44^. We used bioinformatics to computationally model the effect of the mutations made on the AS-IL1α higher-order structure. The RNAsnp web server^45^ predicts the effect of single nucleotide polymorphisms (SNPs) on local secondary structure based on the RNA folding algorithms used in the ViennaRNA package^46,47^. Computational secondary structure analysis revealed that the SNPs made in the IL-1α sequence led to a significant local structural effect (p = 0.0294) on the AS-IL1α lncRNA in the local region of nucleotides 439–509 (horizontal orange bar indicates area of significant structural change) (Fig. 5B). This region of significant structural change is located around the SNP mutations made (black vertical lines). Graphical display of the mutated sequence indicates that there is a disruption in the formation of loops in the RNA structure (Fig. 5C – areas of structural change highlighted in red (IL-1α^mut/mut^) vs. green (IL-1α^+/+^)). This suggests that, although expression of the AS-IL1α lncRNA was not affected by the seven mutations made in the genomic DNA sequence, secondary structure was predicted to be altered, which may be a potential explanation for the loss of IL-1α expression.

## Discussion

We have demonstrated that endogenous, CRISPR-induced point mutations in exon 3 of the *Il1a* gene results in complete loss of inducible expression in mouse BMDMs, BMCs and peritoneal macrophages. We were unable to detect IL-1α protein or mRNA from this mutant allele, indicating a loss of transcriptional regulation.

A recently discovered regulatory lncRNA has been described, which is critical for *Il1a* gene activation. Chan *et al*. demonstrated that AS-IL1α, a lncRNA located on the antisense stand of the *Il1a* gene, is required for the expression of IL-1α. Indeed, when knocked down by shRNA, LPS-induced IL-1α expression is lost^12^. We show that minor changes in the genetic sequence are predicted to result in major lncRNA structural changes, which may explain the complete abrogation of *Il1a* gene expression. Such changes in the secondary structure of the lncRNA could be sufficient to inhibit the recruitment of the transcriptional machinery required for expression of IL-1α thus inhibiting its expression (Fig. 5D).

The observation that in cells heterozygote for the NLS mutations there is a 50% reduction in IL-1α protein and mRNA expression compared to WT cells suggests that the AS-IL1α lncRNA is cis-acting, and directly regulates the IL-1α gene on the sense strand. Should the AS-IL1α act in *trans* we would have expected to have seen partial, if not full, rescue of the mutant allele in heterozygotes. The implications of this discovery are two-fold. Firstly, we show that transcriptional regulation of IL-1α is complex and highly regulated, involving extracellular signaling and intermediate activation of lncRNA regulatory molecules. In mice, immune expression and activity of IL-1α is regulated on multiple levels. Transcription in immune cells can be regulated by NFκB^41^, subcellular localization is regulated by an N-terminal NLS^15^ and activity at the IL-1R receptor is regulated by post-translational modification in the form of processing from a 31 kDa ‘pro’ form to a more biologically active 17 kDa form^48^. Here we suggest that an additional level of regulation, a lncRNA located on the antisense strand^12^, may be extremely sensitive to point mutations through loss of normal secondary structure. This code within a code demonstrates the complexity of cell biology and potentially opens up a new area of research in understanding and defining the numerous additional levels of regulation that may exist in protein expression.

Loss of function of the AS-IL1α lncRNA is not the only possible explanation for the abolition of IL-1α expression. This effect could also be caused by loss of TF binding to enhancer regions located at the mutated site in exon 3. However, Chan *et al*. show that shRNA directed specifically to target AS-IL1α lncRNA expression prevented recruitment of RNA polymerase II to the promotor region of IL-1α at + 22 bp downstream of the transcription start site and is thus critical for the recruitment of transcriptional machinery to this gene^12^. In this experiment from Chan *et al*., the genetic sequence of exon 3 remains unaffected but expression is lost, suggesting that TF recruitment occurs at the promotor regions of *Il1a* in an AS-IL1α-dependent manner. We thus conclude the most likely explanation for loss of IL-1α expression in this CRISPR modified mouse model is the inadvertent perturbation of the structure of the key IL-1α regulator molecule AS-IL1α.

The results from the study also have far-ranging implications in the field of genome engineering. Previously, mutant forms of genes would be typically introduced to cells or mice though exogenous constructs engineered *in vitro*, such as plasmids, viruses or bacterial artificial chromosomes. However, more recently, the ability to directly modify endogenous alleles through gene editing approaches such as ZFNs, TALENs and latterly CRISPR/Cas9 has revolutionized biological research, and potentially medicine. In theory it is now plausible to build a ‘perfect’ representative biological system, where the mutated gene of interest is still controlled by all endogenous regulatory regions and mechanisms. However, this relies on precise knowledge and understanding of the mammalian genome, structure, organisation and gene regulation. In this study our mutations led to an unexpected loss of gene expression. One may envisage a circumstance where such inadvertent effects may not be immediately detected, such as the manipulation of a trans-acting regulatory RNA, cryptic regulatory elements, nested genes and so forth that may have indirect effects on genetic systems. Indeed, non-coding RNAs themselves have been shown in many circumstances to be difficult to target by CRISPR/Cas9, due to their propensity to be found overlapping neighbouring genes^49^.

A common method, also employed in this study, is to include ‘shield’ mutations in DNA repair templates for HDR, to prevent sgRNA/Cas9 complexes re-binding and re-cutting repaired DNA. Typically these mutations target the PAM site or seed sequence of the sgRNA target, and are usually silent or synonymous mutations, changing DNA bases but not the amino acid they encode. However, there is a growing literature of examples where this kind of change has resulted in alterations in mRNA splicing, mRNA folding, stability and regulation of translation, and it is becoming clear that codon usage in different species is not random^50^.

It is likely that in the near future we will see gene editing strategies employed directly in the clinic, where until now the major concerns have been related to off-target effects (OTEs)^51^. We suggest that, especially in therapy, researchers should be cautious about changing the genome with impunity. Some of the newer gene editing strategies developed, such as base editing using deadCas9 or Cas9nickase in conjunction with cytidine deaminase enzyme that does not induce dsDNA breaks, but instead mediates the direct conversion of bases, may mitigate some of these concerns^52^.

CRISPR technology is a powerful weapon in the researcher’s armory, but as we demonstrate, is not without caveats. In this study of the function of IL-1α NLS direct genome engineering has so far proven to be more complicated than expected, and serves as a cautionary notice that any and all implications of genetic modification should be considered. Researchers must be wary of the myriad of unexpected possibilities that may result from making even small changes to the genome.

## Materials and Methods

### Generation of CRISPR reagents and microinjection

For CRISPR targeting we generated sgRNA for each target site according to Shen *et al*.^53^. Briefly, complementary oligos for the sgRNA target sequences were synthesised (Integrated DNA technologies), annealed and ligated into pUC57-sgRNA expression vector. After sequence confirmation plasmids were mini-prepped, linearised with DraI restriction enzyme and used as template in an *in vitro* T7 transcription reaction according to manufacturer’s instructions (NEB). Synthesised sgRNA was purified using Ambion Megaclear kit, eluted in injection buffer (10 mM Tris (pH 7.5), 0.1 mM EDTA (pH 8.0), 100 mM NaCl) and concentration determined by nanodrop. Template DNA was synthesised in a vector (Cellectis) and the 816 bp dsDNA repair region was excised, gel extracted and PCR cleaned, eluted in injection buffer, and concentration determined by nanodrop. Injection mix (final volume 50 μl) was prepared by combining the two sgRNA (final concentration 50 ng μl^−1^ each) with Cas9 protein (labomics, final concentration 100 ng μl^−1^) and incubated at room temperature for 10 min, before adding DNA repair template (final concentration 10 ng μl^−1^). Standard pronuclear microinjection was performed on B6D2F1 (Envigo) hybrid zygotes which led to 2 founder mice that were then back-crossed to C57BL/6 wild-type mice to assess germline penetrance.

### Genotyping and identification of positive offspring

We detected offspring harbouring the desired mutation by amplification with primers Geno F (CCCAAGCAAGGAAAAGGAAGG) and Geno R (GACTGAGTCTTCCCCTCGTA) which target outside the HDR template, and digested with BseRI. BseRI digestion gave preliminary indication of a mutant allele, which was confirmed by pCR-Blunt cloning (Invitrogen) and Sanger sequencing. Two founders, animals 53 and 63, were found to have the mutant allele. It should be noted that in both these animals the second allele had also been targeted by CRISPR/Cas9 and undergone NHEJ repair, resulting in a 59 bp sgRNA to sgRNA deletion and a +11/−15 bp InDel respectively. Animal 53 was used to establish a colony of IL1α NLS mutant mice after confirmation of germline transmission.

### Animals

Animals were maintained under standard laboratory conditions: ambient temperatures of 21 °C (±2 °C), humidity of 40–50%, 12 h light cycle, ad libitum access to water and standard rodent chow. Genotype groups were randomised during the study and experimenters were blinded to genotype during all cell isolation procedures and experiments. All animal experiments were carried out in accordance with the United Kingdom Animals (Scientific Procedures) Act 1986 and approved by the Home Office and the local Animal Ethical Review Group, University of Manchester.

### Cell isolations and assays

#### BMCs

Mouse bone marrow cultures (BMCs) were isolated by flushing marrow from mouse femurs. Red blood cells were lysed by suspending in ACK buffer (Lonza) for 3 min at RT. Immediately after isolation cells were seeded at 5 × 10^6^ cells ml^−1^ in RPMI, 10% FBS, 1% PenStrep, 1% L-glutamine. Cells were treated with LPS (Sigma O26:B6, 1 μg ml^−1^, 3 h) then stimulated with nigericin (Sigma, 10 μM, 1 h), ionomycin (Sigma 10 μM, 1 h) or silica (US Silica, 300 μg ml^−1^, 3 h). Supernatants and lysates were taken and analysed for IL-1α, IL-1β and IL-6 content by ELISA (DuoSet, R&D systems) according to manufacturer’s instructions.

#### Peritoneal macrophages

Peritoneal macrophages were isolated by peritoneal lavage. Mice were anaesthetized with isoflurane (induced at 3–4% in 33% O_2_, 67% NO_2_, maintained at 1–2%) and peritoneal cavities lavaged with 6 ml RPMI. Peritoneal macrophages were counted and seeded at 1 × 10^6^ cells ml^−1^ in 96-well plates in Dulbecco’s Modified Eagle’s Medium (DMEM), 10% fetal bovine serum (FBS, Life Technologies), 100  U ml^−1^ penicillin and 100 μg ml^−1^ streptomycin (PenStrep). Cells were primed with LPS (1 μg ml^−1^, 4 h) then stimulated with ATP (Sigma, 5 mM, 1 h), ionomycin (10 μM, 1 h) or silica (300 μg ml^−1^, 4 h). Supernatants and lysates were taken and analysed for IL-1α, IL-1β and IL-6 content by ELISA (DuoSet, R&D systems) according to manufacturer’s instructions.

#### BMDMs

Mouse bone marrow-derived macrophages were isolated by flushing marrow from mouse femurs. Red blood cells were lysed by suspending in ACK buffer (Lonza) for 3 min at RT. Following lysis, cells were cultured in DMEM, 10% FBS, 1% PenStrep supplemented with 30% L929 mouse fibroblast supernatant conditioned media for 7–10 days. One day prior to experiments, cells were seeded overnight at 1 × 10^6^ cells ml^−1^ in 96-well plates in DMEM, 10% FBS, 1% PenStrep. Cells were primed with LPS (1 μg ml^−1^, 4 h) then stimulated with ATP (5 mM, 1 h), ionomycin (10 μM, 1 h) or silica (300 μg ml^−1^, 4 h). Supernatants and lysates were taken and analysed for IL-1α, IL-1β and IL-6 content by ELISA (DuoSet, R&D systems) according to manufacturer’s instructions.

#### Platelets

Platelet cells were isolated from fresh blood as by Cazneave *et al*.^54^. Briefly, blood was taken via cardiac puncture with acid-citrate-dextrose (ACD) anticoagulant solution at ratio of 1 part ACD to 5 parts blood. Anticaogulated blood was centrifuged at 2300 x g for 45 seconds and the platelet-rich plasma (PRP) taken. PRP was then centrifuged at 2200 x g for 2 min and the platelet-containing pellet lysed with Triton-X solution. Lysates were corrected for total protein using a BCA assay (ThermoFisher) according to manufacturer’s instructions and analysed for IL-1α content by ELISA (DuoSet, R&D systems).

### qPCR

BMDMs were treated with vehicle or LPS (1 μg ml^−1^, 5 h) lysed in TRIzol Reagent (ThermoFisher) and RNA isolated according to the manufacturer’s instructions. RNA (0.6 μg) was converted to cDNA using SuperScript™ III Reverse Transcriptase (ThermoFisher) according to manufacturer’s instructions. qPCR was performed using Power SYBR® Green PCR Master Mix (ThermoFisher) in 384-well format using an 7900HT Fast Real-Time PCR System (Applied Biosystems). 16.6 ng cDNA was loaded with 5 pmol primer/well in triplicate. Data were normalized to the expression of the housekeeping gene 18 S rRNA. Primers used were: IL-1α Forward - TCTCAGATTCACAACTGTTCGTG, IL-1α Reverse - AGAAAATGAGGTCGGTCTCACTA, AS-IL1α Forward-AGGCTTGGGATTCACTTGAC, AS-IL1α Reverse –TCTCTCTGGGCTTCAGTTCC, 18 S rRNA Forward – CGCGGTTCTATTTTGTTGGT, 18 S rRNA Reverse – AGTCGGCATCGTTTATGGTC. Data were analysed using the relative standard curve method.

### Western blotting

Western blot analysis was performed on supernatants and lysates for IL-1β and IL-1α. Samples were run on 12% sodium dodecyl sulphate (SDS) polyacrylamide gels. Gels were transferred at 15 V onto nitrocellulose membrane (GE Life Sciences) using a Trans-Blot SD semi-dry transfer system (Bio-Rad) before blocking with 5% w/v milk in phosphate-buffered saline, 1% Tween 20 (Sigma) (PBST) for 1 h at RT. Membranes were washed and incubated (4 °C) overnight in goat anti-mouse IL-1β (100 ng ml^−1^, R&D Systems) or goat anti-mouse IL-1α (1 μg ml^−1^, R&D Systems) primary antibody in PBST, 0.1% bovine serum albumin (BSA). Following this, membranes were washed and incubated with rabbit anti-goat (550 ng ml^−1^) secondary antibody (Dako) in PBST, 5% milk for 1 h at RT. Finally, membranes were washed and incubated in Amersham ECL Western Blotting Detection Reagent (GE Life Sciences) before exposure using a G:BOX gel doc system (Syngene).

### Flow cytometry

Bone marrow cells were isolated by flushing and lysed with ACK lysis buffer (Lonza). Splenocytes were prepared by homogenising whole spleens and passing through 70 μm cell strainers before ACK lysis. Cells were stained with antibodies consisting panels to detect neutrophil/monocyte populations (CD45 (Tonbo), CD11b (eBioscience), CD115 (Biolegend), Ly6C and Ly6G (Tonbo)) and T and B-cell populations (CD8 (eBioscience), CD4 (Biolegend), CD19 (Biolegend), TCRβ (Biolegend)) for 25 min on ice in the dark before washing twice and fixing in paraformaldehyde (2% in PBS) for 15 min at RT. Dead cells were excluded by use of a Zombie NIR Live/Dead fixable dye (Biolegend). The following day, flow cytometry was performed using a FACSCanto flow cytometer (BD Biosciences) and analysed with FlowJo software (FlowJo LLC).

### RNA structure prediction

The nucleotide sequence of AS-IL1α (Accession - KR095173) was inputted to the RNAsnp web server along with the 7 SNPs made (G399U-A441G-C442G-U443A-C444G-U455C-U457C). The ‘mode 2’ algorithm was selected to search for significant structural effects of SNPs on large RNA sequences (>1000 nt) using the local folding method RNAplfold.

### Statistical analyses

Data are presented as mean values + standard error of the mean (s.e.m). Levels of significance were P < 0.05 (*), P < 0.01 (**), P < 0.001 (***). Statistical analyses were carried out using GraphPad Prism (version 7). Cytokine production and secretion were analysed with a two-way ANOVA with repeated measures followed by Tukey’s post-hoc comparisons. IL-1α in platelets was measured by a one-way ANOVA with Tukey’s post-hoc comparisons. qPCR data were analysed using a two-way ANOVA with repeated measures followed by Tukey’s post-hoc comparisons. Transformations were applied where necessary. lncRNA secondary structure was analysed using the RNAplfold algorithm^47^.

### Data availability

The data that support the findings of this study are available from the corresponding author on request.

## Electronic supplementary material

Supplementary Information
