# Supplementary material for: CRISPR/Cas9 mediated mutation of mouse IL-1α nuclear localisation sequence abolishes expression
Source: Sci Rep. 2017 Dec 6;7:17077. doi: 10.1038/s41598-017-17387-x (PMC5719027; doi:10.1038/s41598-017-17387-x)
Supplement: Supplementary file 1 — Supplementary Information [file 41598_2017_17387_MOESM1_ESM.pdf]

# SUPPLEMENTARY INFORMATION

## CRISPR/Cas9 mediated mutation of mouse IL-1α nuclear localisation sequence abolishes expression

Michael J. D. Daniels[1], Antony D. Adamson[2], Neil Humphreys[2], David Brough[1]*

[1]Division of Neuroscience and Experimental Psychology, School of Biological Sciences, Faculty of Biology, Medicine and Health, Manchester Academic Health Science Centre, University of Manchester, AV Hill Building, Oxford Road, Manchester, M13 9PT, U.K.

[2]Transgenic unit, Faculty of Biology, Medicine and Health, University of Manchester, AV Hill Building, Oxford Road, Manchester, M13 9PT, U.K.

*To whom correspondence should be addressed: David Brough,* Division of Neuroscience and Experimental Psychology, School of Biological Sciences, Faculty of Biology, Medicine and Health, Manchester Academic Health Science Centre, University of Manchester, AV Hill Building, Oxford Road, Manchester, M13 9PT, U.K. *Tel: +44 (0)161 275 5039. Fax: +44 161 275 5948. Email: david.brough@manchester.ac.uk.*

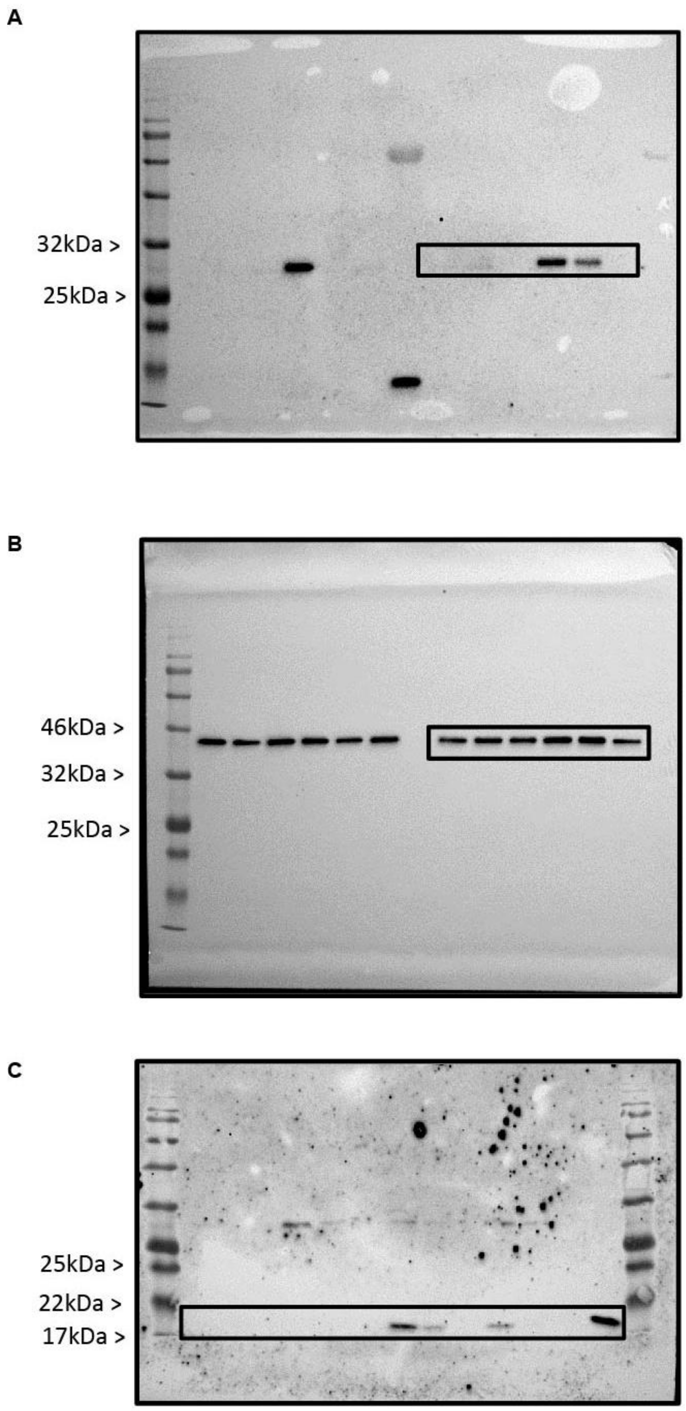


**Supplementary Figure 1:** Full length blots from figure 3. Blots showing IL-1α and β-actin in BMDM lystates from Fig. 3G (A, B) and IL-1α BMDM supernatants from Fig. 3H (C).
